# The psychosocial context of chronic pain in people living with HIV

**DOI:** 10.1097/PR9.0000000000000721

**Published:** 2019-03-14

**Authors:** Whitney Scott

**Affiliations:** Health Psychology Section, Institute of Psychiatry, Psychology, and Neuroscience, King's College London, London, United Kingdom

**Commentary on:** Penn TM, Trost Z, Parker R, Wagner WP, Owens MA, Gonzalez CE, White DM, Merlin JS, Goodin BR. Social support buffers the negative influence of perceived injustice on pain interference in people living with HIV and chronic pain. PAIN Rep 2019:e710. doi: 10.1097/PR9.0000000000000710.

The psychosocial context is integral to the experience and treatment of pain: “[pain] is a distressing experience associated with actual or potential tissue damage with sensory, emotional, cognitive, and social components.”^30^ The social context influences exposure to risk of developing chronic pain and conditions that can cause pain, such as HIV.^1,26^ Characteristics of the social environment, such as invalidating or stigmatising responses from others, are associated with worse pain outcomes.^2,3^ Macrolevel social factors, such as the organization of health care systems and disability benefit policies, also impact the lives of people with chronic pain.^13^ Social context modulates pain experience and, in turn, pain threatens social inclusion.^10^ There are complex interactions between the social environment and an individual's cognitive, affective, and behavioural responses to pain.^8^

The study by Penn et al. in this issue brings needed attention to the psychosocial context of chronic pain in people living with HIV (PLWH) in the current treatment era.^17^ As PLWH age and have normal life expectancy with antiretroviral therapy (ART), quality of life is now a focus.^9,15^ Chronic pain, as highlighted by Penn et al., is a major threat to functioning and quality of life in PLWH. Randomised controlled trials have shown that analgesic medications have poor efficacy for treating common pain conditions in PLWH, such as painful peripheral neuropathy.^4,19^ Better understanding of psychosocial processes that influence the experience of chronic pain in this population can inform the development of more holistic treatments.

The focus on social support in the study by Penn et al. is welcome, as social factors have received relatively limited attention in the literature on chronic pain in PLWH. In a recent systematic review of psychosocial factors associated with chronic pain outcomes in this population, only 4 of the 46 included studies investigated social processes such as social support and stigma.^20^ In their cross-sectional, questionnaire-based study, Penn et al. found that perceptions of pain-related injustice (ie, appraisals of loss, unfairness, and blame) were associated with greater pain interference in PLWH; however, high levels of social support attenuated this association.^17^ Although they did not directly assess stigma, the authors argue that stigma related to HIV and the pain itself, particularly when not attributed to clear pathology, might give rise to appraisals of one's situation as unjust.^17^

Following from their findings, Penn et al.^17^ identify that efforts to enhance social support may improve pain outcomes among people with HIV and chronic pain. In particular, they suggest that fostering a strong therapeutic alliance may help to mitigate the impact of low social support.^17^ The therapeutic alliance is of course important for improving pain management outcomes.^7^ There is also preliminary evidence that perceived injustice is associated with disruptions in the working alliance among people with chronic pain attending multidisciplinary rehabilitation.^22^ However, further consideration for how to bolster social support in the daily lives of PLWH and chronic pain is needed.

Stigma associated with HIV may understandably limit a person's willingness to seek out support for managing pain and HIV outside of the healthcare context. People living with HIV may conceal pain from others to avoid revealing their HIV status.^27,28^ It is plausible that unwillingness to disclose pain is particularly likely for pain attributed to HIV or ART (eg, painful peripheral neuropathy) that may be more difficult to explain than more “normal” aches and pains, such as low back pain. Anticipatory stigma may lead to reduced opportunities to elicit social support.^5^ Compassion-focussed techniques that help people respond more effectively to emotions associated with stigma and injustice, such as shame and anger, may address these barriers to social support.^24^

Penn et al.^17^ importantly highlight the nature of intersecting stigmatized identities, which may have contributed to injustice perceptions in their sample. Notably, their sample may have experienced stigma and injustice in relation to HIV and pain, as well as their mental health, race, and socioeconomic status. Therefore, psychosocial approaches for pain management in PLWH may need to be modified to meet these intersecting needs, so existing disparities are not exacerbated. The LAMP trial is an important example of tailoring cognitive-behavioural therapy for pain management to address the needs of participants with chronic pain who were multiply disadvantaged in terms of race, income, and education level.^25^ The trial evaluated a literacy-adapted cognitive-behavioural treatment and found improved pain outcomes compared with usual care.^25^ Different modes of delivering psychosocial pain management for PLWH may be necessary given differences in demographics and socioeconomic status across contexts.^17,18,27^

The LAMP trial is also notable for the way in which the researchers established strong collaborations with community partners, which facilitated study recruitment and retention.^6^ Indeed, the use of peers to facilitate delivery of psychosocial treatments has shown promise for improving quality of life in PLWH.^23^ The ongoing STOMP trial^14^ described by Penn et al. that incorporates peer support into a behavioural pain management treatment for PLWH will be a significant contribution to the field.

A multifaceted approach that fosters capacities in the individual to respond more effectively to adversity and that targets systemic injustices that detrimentally impact pain outcomes is needed.^29^ The CHAMP study is an innovative example of such an approach.^12^ CHAMP examined a combined intervention based on Acceptance and Commitment Therapy and social justice capacity building in PLWH. The Acceptance and Commitment Therapy component aimed to increase psychological flexibility to respond more helpfully to internalised stigma; the social justice component used collaborative learning to enhance health literacy, systemic advocacy, and community mobilization. Crucially, the intervention connected PLWH from racial/ethnic minority groups and HIV-negative community leaders from faith-based, media/arts, and social justice sectors. Although preliminary and not focused on pain management, the results show promise for reducing stigma and increasing tendencies for social justice advocacy.^12^ A similar approach may be useful to address injustice, stigma, social support, and pain management in PLWH.

In the study by Penn et al., participant age was not significantly correlated with perceived injustice, social support, or pain intensity/interference. However, there may be important cohort effects that impact the psychosocial context of pain management in PLWH. For example, there is a cohort of people who lived through the early days of the HIV/AIDS epidemic and witnessed the shift from HIV as a terminal to chronic illness. These experiences may influence the extent to which HIV and pain are experienced with a sense of unfairness and loss, and how this cohort engages with social support compared with those diagnosed more recently in the epidemic.^16^ In addition, before the widespread availability of ART, a palliative care approach to pain management was used, and high-dose opioids were common.^11^ Thus, a more holistic biopsychosocial approach to pain management may reflect a paradigm shift for that cohort. There is promising evidence of successful opioid reduction in this cohort through collaborative, person-centred interdisciplinary engagement.^11^

Sensitivity in language is needed when discussing injustice and social inequities. Descriptions of pain-related injustice perceptions as “harmful” and “maladaptive” are problematic. The use of pathologizing language to refer to experiences, which may be rooted in actual systemic injustice, may inadvertently perpetuate stigma and injustice.^10^ Rather than evaluating injustice appraisals as “maladaptive,” it may be more useful to identify specific instances when one's behavioural response to injustice is more or less helpful in terms of one's life values.^21^ We must also ensure that we do not overattribute responsibility for pain-related injustice to people living with pain; this may detract focus from efforts to create more equitable social systems to improve the lives of stigmatised and marginalised groups.

## Disclosures

The author has no conflict of interest to declare.
